# Adherence to endocrine therapy and survival outcomes in hormone receptor–positive breast cancer

**DOI:** 10.3389/fonc.2026.1824288

**Published:** 2026-05-01

**Authors:** Xiao Ju, Ke Han

**Affiliations:** 1 Department of Radiation Oncology and Shandong Provincial Key Laboratory of Radiation Oncology, Shandong Cancer Hospital and Insititute, Shandong First Medical University and Shandong Academy of Medical Sciences, Jinan, Shandong, China; 2 Department of General Surgery, Jinan Fifth People’s Hospital Affiliated to Shandong Second Medical University, Jinan, Shandong, China

**Keywords:** adherence, adjuvant endocrine therapy, competing risk, hormone receptor–positive breast cancer, overall survival, trajectory analysis

## Abstract

**Objective:**

To evaluate the association between adherence to adjuvant endocrine therapy and overall survival as well as breast cancer–specific survival in patients with hormone receptor–positive breast cancer, to characterize the dose–response and dynamic trajectory features of adherence, and to explore the potential mediating roles of treatment interruption and subsequent treatment escalation.

**Methods:**

This single-center real-world cohort study enrolled patients with hormone receptor–positive breast cancer who underwent surgery and initiated adjuvant endocrine therapy. Adherence was assessed during the first 12 months following treatment initiation using a prespecified landmark design to mitigate time-related biases. The primary exposure, proportion of days covered, was analyzed both as a dichotomized variable for stratified comparisons and as a continuous variable for dose–response and non-linear modeling; longitudinal trajectory analysis was further applied to identify distinct long-term adherence behavioral phenotypes. The primary outcome was overall survival, and the secondary outcome was breast cancer–specific survival. Propensity score weighting was employed to balance baseline characteristics, and competing risk regression was used for cause-specific mortality. Mediation analysis was conducted to quantify the indirect effects of treatment interruption and subsequent treatment escalation in the adherence–prognosis pathway.

**Results:**

Over the follow-up period, high adherence was significantly associated with superior overall survival, an association that remained robust after propensity score weighting. Competing risk analysis demonstrated that high adherence was also associated with a reduced risk of breast cancer–specific mortality. Dose–response analysis revealed a non-linear relationship between adherence and mortality risk, with the steepest risk reduction observed in the moderate adherence range. Trajectory analysis identified distinct patterns of adherence—including persistently high, gradual decline, and persistently low—with the rapid-decline and persistently low trajectories conferring the highest risk of adverse outcomes. Mediation analysis indicated that treatment interruption and subsequent treatment escalation partially explained the association between adherence and survival.

**Conclusion:**

In this real-world population, adherence to adjuvant endocrine therapy is closely associated with survival outcomes in hormone receptor–positive breast cancer, exhibiting dose–response and dynamic trajectory characteristics. Conceptualizing adherence as a continuous, time-varying behavioral phenotype may enable more precise identification of at-risk populations and inform optimization of long-term management strategies.

## 1 Introduction

Early-stage breast cancer remains one of the most commonly diagnosed malignancies among women globally, with its epidemiological burden continuing to escalate across regions. According to the latest estimates from GLOBOCAN 2022, approximately 2.30 million new cases and 666,000 deaths due to female breast cancer occurred worldwide in 2022, rendering it a critical public health challenge with profound implications for female life expectancy, health resource allocation, and socioeconomic systems (1, 2). Global trend analyses further indicate that breast cancer incidence continues to rise in the majority of countries, and against a backdrop of population aging and shifting lifestyle patterns, the absolute number of new cases is projected to increase substantially by 2050. This trajectory underscores the urgent need to strengthen long-term disease management and risk mitigation strategies (3). From a molecular subtype perspective, early-stage HR+ disease constitutes the largest proportion of all breast cancers and represents the predominant population in routine clinical care. Given that HR+ is the most common molecular subtype, defining its optimal treatment and follow-up strategies is numerically essential for improving population-level outcomes, especially as these patients require distinct management of late recurrence risk and extended oral therapies (4, 5).

For patients with early-stage HR+ breast cancer, adjuvant endocrine therapy (AET) is a central component of postoperative systemic treatment and plays an essential role in reducing the risk of disease recurrence and improving long-term survival (6). Contemporary clinical practice guidelines recommend that patients with early-stage HR+ breast cancer receive at least five years of AET, with extension to 7–10 years considered on the basis of individual recurrence risk, treatment tolerance, and anticipated clinical benefit, in order to further mitigate late relapse events (7). This therapeutic paradigm is grounded in the well-documented “late recurrence” phenotype of HR+ tumors, wherein a persistent hazard of recurrence extends well beyond five years post-diagnosis, rendering sustained adherence to AET a critical determinant of long-term benefit (8). Nevertheless, in contrast to the highly controlled setting of randomized trials, real-world adherence to long-term endocrine therapy declines considerably, with a substantial subset of patients discontinuing or interrupting treatment early, thereby attenuating the population-level effectiveness of an otherwise efficacious intervention (9). A recent systematic review of real-world evidence further demonstrates that adherence and persistence follow a time-dependent decline, and that suboptimal adherence is robustly associated with inferior prognostic outcomes, highlighting the optimization of long-term medication-taking behavior as a potentially modifiable lever for improving survival in HR+ breast cancer (10).

Poor adherence to endocrine therapy arises from a multifactorial etiology, among which treatment-related adverse effects and impaired quality of life are consistently identified as the most proximate and influential determinants (11). In the protracted course of HR+ breast cancer management, drug toxicities not only compromise daily functional status but may also erode patients’ willingness and capacity to maintain long-term therapy. Taking aromatase inhibitor (AI)–related musculoskeletal symptoms as an illustrative example, recent clinical evidence indicates a high incidence of this adverse effect and a significant association with treatment discontinuation, lending support to a mechanistic sequence linking therapeutic toxicity to reduced adherence and, ultimately, unfavorable prognosis (12). Concurrently, adherence should not be conceptualized as a static, binary exposure; rather, it exhibits pronounced heterogeneity across patient populations. A recent longitudinal real-world study focusing on premenopausal HR+ breast cancer patients revealed that adherence follows a dynamic trajectory shaped by disease risk characteristics, comorbidity burden, and concomitant treatments, suggesting that adherence is a complex, time-dependent construct (13). Moreover, the impact of adherence on clinical outcomes is not solely contingent on whether treatment is maintained, but may also be closely tied to phenotypic features of adherence behavior—such as the intensity of adherence, the rate of decline, and the timing of treatment interruptions (14). Trajectory-based analyses of large real-world cohorts have further identified distinct patterns of adherence evolution (e.g., persistently high adherence, gradual decline, and persistently low adherence), with these trajectory classes demonstrating systematic associations with demographic profiles, disease stage, and therapeutic regimens. These findings provide both theoretical rationale and empirical direction for risk-stratified, precision-oriented intervention strategies (15).

Although a substantial body of observational research has demonstrated a significant association between adherence to AET and survival benefits, the current evidentiary landscape remains constrained by several key methodological challenges and structural shortcomings that may compromise the validity and robustness of causal inference. Adherence, by nature a time-dependent exposure, is susceptible to time-related structural biases—most notably immortal time bias, a phenomenon in observational studies where a period of follow-up is incorrectly assigned to an exposure group because the individual had to remain alive (and thus ‘immortal’) during that interval to fulfill the criteria for being ‘exposed’ (16, 17). This bias arises when patients must survive long enough to meet a certain adherence threshold within the assessment window; since those who die early are systematically excluded from the ‘adherent’ group, the adherent group is granted ‘immortal’ status during that period, thereby falsely inflating the apparent protective effect of adherence (16). Recent discourse in clinical epidemiology regarding the target trial framework has underscored the importance of explicitly defining time zero, treatment strategies, and follow-up windows to emulate the design principles of a randomized trial, thereby mitigating time-varying confounding and selection bias (18). In parallel, much of the extant literature continues to rely on dichotomous classifications of adherence based on fixed thresholds (e.g., proportion of days covered or medication possession ratio ≥80%), emphasizing associational comparisons while offering limited insight into the continuous dose–response relationship, potential non-linear threshold effects, and long-term dynamic trajectories of adherence. This analytic approach curtails the capacity to accurately characterize the true functional form of the exposure–outcome relationship (17). At the outcome level, time-to-event endpoints such as breast cancer–specific mortality frequently co-occur with competing events—most notably non-cancer deaths. Failure to appropriately account for competing risks using methods such as the Fine–Gray subdistribution hazard model may result in biased risk estimates and misinterpretation of effect sizes (19). Furthermore, real-world data are replete with complex confounding structures and incomplete measurement of confounders; in the absence of rigorous covariate development, propensity score methods, and multi-faceted sensitivity analyses, the causal interpretability of findings remains tenuous. Accordingly, there is a pressing need to strengthen internal validity and external generalizability through more robust design frameworks and statistical approaches (20).

In light of the theoretical and empirical considerations outlined above, the present study was designed to systematically evaluate the association between adherence to adjuvant endocrine therapy and survival outcomes in hormone receptor–positive breast cancer within a real-world cohort framework, while reinforcing the rigor and interpretability of causal inference at both the design and analysis stages. We predefined the start of follow-up and employed a landmark approach to attenuate the influence of time-related biases on effect estimates. Propensity score weighting techniques were implemented to improve baseline covariate balance and enhance intergroup comparability. Outcome assessment incorporated both overall survival and breast cancer–specific survival within a competing risks framework to improve the accuracy and clinical interpretability of risk estimates. With respect to exposure modeling, adherence was operationalized not only as a categorical stratification variable but also as a continuous measure to evaluate dose–response and potential non-linear relationships; in addition, trajectory-based methods were employed to delineate the dynamic evolution of long-term adherence phenotypes. The study further explored the mediating roles of treatment interruption and subsequent treatment intensification in the adherence–prognosis pathway, aiming to elucidate potential behavioral–clinical mechanisms. By constructing an analytic framework that explicitly accounts for dynamic adherence trajectories and competing mortality risks, this investigation seeks to generate translationally relevant evidence to inform the optimization of long-term management strategies, the identification of windows for adherence intervention, and the precision allocation of health care resources in HR+ breast cancer survivorship care.

## 2 Materials and methods

### 2.1 Patient characteristics

#### 2.1.1 Study design

This investigation employed a retrospective cohort design, with the study cohort constructed using data derived from an electronic health record (EHR) system, a pharmacy prescription database, and a longitudinal follow-up registry.

The study population was drawn from the institutional tumor registry of a tertiary cancer specialty hospital, which integrates multidimensional clinical information from outpatient visits, inpatient hospitalizations, pharmacy dispensing records, and regional death registries. The enrollment period spanned from January 1, 2021, through December 31, 2024, and included patients who had received a first-time diagnosis of breast cancer and undergone curative-intent surgery. All patients meeting eligibility criteria entered the cohort at the index date, defined as the date of their first documented dispensing of adjuvant endocrine therapy. Follow-up continued until the occurrence of death, loss to follow-up, or the administrative censoring date of December 31, 2025, whichever occurred first. A total of 612 patients with breast cancer were initially screened. Following rigorous application of predefined inclusion and exclusion criteria, 318 patients with hormone receptor–positive disease who received adjuvant endocrine therapy and had complete prescription records were included in the final analytic cohort.

To mitigate the risk of immortal time bias, a 12-month landmark design was implemented. The adherence assessment window was defined as the 12-month period immediately following the index date. Only patients who remained alive, had not experienced the study endpoint, and had evaluable adherence data during this window were carried forward into the survival analysis. This study was designed, conducted, and reported in strict adherence to the Strengthening the Reporting of Observational Studies in Epidemiology (STROBE) guidelines.

#### 2.1.2 Ethical approval and informed consent

This study was reviewed and approved by the Institutional Review Board of Jinan Fifth People’s Hospital Affiliated to Shandong Second Medical University. Given the retrospective nature of the analysis, which relied exclusively on data collected during routine clinical care and entailed no additional interventions or procedures involving patients, and given that all data underwent de-identification and anonymization prior to statistical analysis, the requirement for written informed consent was waived by the ethics committee. The study was conducted in full accordance with the principles articulated in the Declaration of Helsinki. Data security measures—including tiered access authorization, encrypted storage, and an audit trail system—were implemented to safeguard patient privacy and data integrity.

### 2.2 Eligibility criteria

#### 2.2.1 Inclusion criteria

Patients were eligible for inclusion if they satisfied all of the following conditions:

1. Histopathologically confirmed primary breast cancer;
2. Immunohistochemical evidence of estrogen receptor (ER) and/or progesterone receptor (PR) positivity, defined as nuclear staining in ≥1% of tumor cells;
3. Clinical stage I–III disease (American Joint Committee on Cancer [AJCC], version as specified);
4. Underwent curative-intent surgical resection;
5. Received adjuvant endocrine therapy (tamoxifen, an aromatase inhibitor, or a sequential regimen) postoperatively;
6. Had complete prescription records sufficient for the calculation of medication adherence;
7. Completed at least 12 months of follow-up.

#### 2.2.2 Exclusion criteria

Patients meeting any of the following criteria were excluded from the study:

1. *De novo* stage IV metastatic breast cancer at initial diagnosis;
2. Received neoadjuvant endocrine therapy with an indeterminate treatment start date;
3. Missing critical clinical information (e.g., disease stage, histologic grade, or key treatment modalities);
4. Insufficient prescription records to compute adherence (e.g., only a single dispensing event documented);
5. Developed a second primary malignancy during the follow-up period;
6. Died or were lost to follow-up during the adherence assessment window (applicable to the landmark analysis).

### 2.3 Study procedures

#### 2.3.1 Data extraction and management

An initial case inventory was generated from the institutional cancer registry. Specifically, breast cancer diagnosis records from January 1, 2021, to December 31, 2024, were retrieved to extract unique patient identifiers, which were then cross-referenced with pathology records to ascertain hormone receptor status. Eligible cases were subsequently linked to surgical records and pharmacy dispensing data to confirm receipt of curative-intent surgery and adjuvant endocrine therapy, thereby forming the preliminary screening cohort.

Following case identification, the authors, working independently, systematically extracted demographic information, tumor pathological characteristics, treatment details, prescription records, and follow-up data from the EHR, pathology database, treatment management system, pharmacy system, and follow-up registry, guided by a pre-specified data dictionary. All date fields were uniformly formatted (YYYY-MM-DD); categorical variables were standardized according to a consistent coding scheme; continuous variables were retained as original values with measurement units recorded.

Raw data derived from disparate sources were merged via one-to-one matching on the unique patient identifier to construct the raw analytic database. A comprehensive log file was maintained throughout the data integration process, documenting rates of missingness, duplicate records, and out-of-range values to ensure full traceability of all data-handling procedures.

To ensure data quality, dual independent verification was performed for all key variables—including date of diagnosis, date of surgery, hormone receptor status, date of endocrine therapy initiation, and date of death. Discrepancies were adjudicated through consensus by consulting the original source system and documenting the rationale for the final determination. Records with implausible values were individually traced, verified, and either corrected or excluded.

Prior to statistical analysis, all data underwent de-identification; personally identifiable information was removed and replaced with a unique study identification number. De-identified data were stored on an encrypted server accessible only to authorized study personnel.

#### 2.3.2 Operationalization of adherence to endocrine therapy

All adjuvant endocrine therapy prescription records were extracted from the institutional pharmacy management system. Retrieved data fields included prescription date, dispensing date, drug name, strength and dosage form, quantity dispensed, and days of supply. Only records with a dispensed or picked-up status were retained; records indicating medication return or cancellation were either corrected or excluded based on system annotations. All drug names were mapped to standardized therapeutic categories using institutional formularies or the Anatomical Therapeutic Chemical (ATC) classification system.

The index date was defined as the date on which a patient first picked up a dispensed adjuvant endocrine therapy post-surgery. In the event of multiple prescription records with identical dispensing dates, the record with the longer days of supply was prioritized; if days of supply were identical, the record with the earliest system timestamp was selected. Instances where the prescription date preceded the date of curative surgery were retrospectively reviewed to determine whether the prescription corresponded to neoadjuvant therapy; such cases were handled in accordance with the prespecified exclusion criteria.

To compute adherence, a day-level medication coverage calendar was constructed for each patient using the Proportion of Days Covered (PDC). The PDC was operationalized as the total number of unique days for which the medication was available, divided by the number of days in the 12-month landmark assessment window (PDC = $\frac{\text{Days Covered}}{365}\times 100\%$), effectively capturing the continuity of drug availability. For each dispensing event, the dispensing date was designated as the start of a coverage episode spanning the days of supply provided. In cases where overlapping coverage intervals resulted from early refills, the coverage start date for the subsequent dispensing was shifted forward to the day immediately following the end of the previous coverage period, thereby avoiding double counting. If a patient switched between endocrine therapy agents during the assessment window, any day covered by any dispensed agent was counted as a covered day.

A 12-month landmark design was implemented, with the adherence assessment window defined as the 365 consecutive days immediately following the index date. Only patients who survived, did not experience the study endpoint, and had complete prescription data during this window were included in subsequent survival analyses. The landmark time point was defined as day 365 post-index date, and all survival analyses were conducted with time-at-risk commencing at this point. Following completion of adherence calculations, separate datasets were constructed: a prescription-level adherence dataset and an analysis-ready landmark dataset.

#### 2.3.3 Follow-up data integration and event verification

Follow-up information was primarily obtained from the institutional follow-up management system, outpatient visit records, and inpatient discharge summaries. The date of last follow-up, survival status, and method of follow-up were initially extracted for each patient. For those with incomplete follow-up records, outpatient revisit logs and inpatient medical records were retrospectively reviewed to ascertain the most recent documented healthcare contact; this date was used to determine the censoring time.

All study subjects were subsequently matched to the institutional death registry to retrieve dates and, where available, causes of death. In cases where no death event was recorded in institutional systems, supplemental queries were submitted to regional death registries. Discrepancies in death information across data sources were resolved by reviewing death certificates or end-of-life medical records, and the basis for adjudication was recorded.

Following multi-source integration and verification, a final follow-up database was constructed and subsequently merged with adherence assessment data and clinical characteristic data to support survival analysis.

#### 2.3.4 Covariate construction for confounding control

Covariates included in the analysis were developed in accordance with a pre-specified data extraction protocol. Tumor stage was determined by synthesizing clinical records, imaging studies, and surgical pathology reports; histologic grade, HER2 status, and Ki-67 labeling index were primarily derived from pathology reports; in the event of multiple test results for the same patient, the results from the initial surgical specimen were prioritized. Treatment-related variables—including chemotherapy, radiotherapy, targeted therapy, and the specific endocrine regimen—were ascertained from physician order entry records and treatment registration systems, and were uniformly coded as binary or categorical variables.

Comorbidity burden was quantified using the Charlson Comorbidity Index (CCI). Inpatient and outpatient diagnosis codes recorded during the 12-month period preceding the index date were extracted and mapped to the CCI scoring system using a standardized algorithm. Each distinct comorbidity was counted only once, yielding a total CCI score for each patient.

All time-related variables were subjected to consistency checks to ensure temporal logic among date of diagnosis, date of surgery, date of treatment initiation, landmark time point, and date of follow-up termination. Records exhibiting temporal anomalies were individually traced, verified, and corrected. After completion of these procedures, a final covariate dataset was generated for statistical analysis.

### 2.4 Outcome measures

#### 2.4.1 Primary outcomes

##### 2.4.1.1 Overall survival

Overall survival was defined as the time interval from the landmark time point to death from any cause, expressed in years. For patients who remained alive or were lost to follow-up prior to the administrative censoring date, survival time was censored at the date of last documented contact. OS, as a global indicator of patient prognosis, was employed to evaluate the comprehensive impact of adherence to endocrine therapy on long-term survival outcomes. In statistical analyses, OS was modeled as a time-to-event variable; descriptive analyses compared survival status across adherence groups over the follow-up period.

##### 2.4.1.2 Breast cancer–specific survival

Breast cancer–specific survival was defined as the time interval from the landmark time point to death attributed to breast cancer. Cause of death information was primarily derived from death registry data and end-of-life medical records, with classification based on International Classification of Diseases (ICD) codes. Deaths due to non-breast cancer causes were treated as competing events in the analysis. BCSS was used to isolate the specific effect of adherence on breast cancer–related mortality, thereby minimizing the attenuating or distorting influence of non-cancer deaths on interpretation.

#### 2.4.2 Secondary outcomes and scoring systems

##### 2.4.2.1 Adherence dose–response score

Adherence level was quantified as a continuous variable using the proportion of days covered (PDC). A categorical stratification variable was also constructed for risk stratification. Based on prior literature and clinical considerations, PDC was categorized into four ordinal levels: very high adherence (≥90%), high adherence (80%–89%), moderate adherence (60%–79%), and low adherence (<60%). The continuous PDC variable was used to evaluate linear or non-linear associations between incremental changes in adherence and survival outcomes, while the categorical variable facilitated comparisons of risk gradients across adherence strata, enabling exploration of potential dose–response patterns and threshold effects.

##### 2.4.2.2 Longitudinal adherence trajectory score

PDC values were calculated over successive time intervals during the assessment window and subsequent follow-up, generating a longitudinal sequence of adherence measures for each patient. Using trajectory classification methods, patients were categorized into distinct adherence patterns: persistently high adherence, gradual decline, fluctuating adherence, and persistently low adherence. Persistently high adherence was defined as PDC consistently ≥80% throughout follow-up; gradual decline as initial PDC ≥80% with a subsequent decline of ≥20%; fluctuating adherence as repeated oscillations in PDC of ≥20%; and persistently low adherence as PDC <80% throughout the entire observation period. Each patient was assigned to a trajectory class based on their predominant adherence pattern; these classes were used to examine associations between long-term medication-taking behavioral phenotypes and survival outcomes.

##### 2.4.2.3 Treatment-stratified adherence effect score

Patients were stratified according to the specific endocrine regimen received: tamoxifen monotherapy, aromatase inhibitor monotherapy, or sequential therapy. Within each treatment subgroup, adherence was dichotomized using the conventional threshold of PDC ≥80% versus <80%, and the strength of the association between adherence and survival outcomes was estimated separately. By comparing the magnitude of adherence effects across different therapeutic strategies, this scoring system aimed to elucidate the potential modifying role of drug class in the adherence–prognosis pathway.

##### 2.4.2.4 Early treatment interruption and discontinuation score

Medication interruption was operationalized based on gaps in prescription coverage, with the length of continuous uncovered intervals serving as the core criterion. Interruptions were graded as follows: no significant interruption (<30 days), mild interruption (30–59 days), moderate interruption (60–89 days), and severe interruption (≥90 days). Permanent discontinuation was defined as a gap of ≥180 consecutive days without any documented dispensing. Patients experiencing a moderate or severe interruption within 24 months of the index date were classified as exhibiting early treatment interruption. This graded classification system was used to quantify the severity of non-persistence and to evaluate its association with survival outcomes.

##### 2.4.2.5 Composite progression risk score

A composite endpoint capturing the overall burden of disease progression was constructed, encompassing breast cancer–specific death, development of distant metastasis, and initiation of second-line systemic therapy. Each event contributed one point to the total score, which ranged from 0 to 3 per patient. Based on their total score, patients were categorized into low-risk (0 points), low-intermediate risk (1 point), high-intermediate risk (2 points), and high-risk (3 points) strata. This composite score was designed to evaluate the impact of adherence on the full spectrum of disease progression.

##### 2.4.2.6 Adherence–persistence composite score

A composite measure integrating adherence and persistence was developed. Adherence was dichotomized as PDC ≥80% (1 point) versus <80% (0 points). Persistence was dichotomized as continued endocrine therapy without permanent discontinuation for ≥36 months (1 point) versus <36 months (0 points). The sum of these two binary indicators yielded a composite score ranging from 0 to 2, based on which patients were classified as optimal adherence-persistence, intermediate, or poor adherence-persistence groups. This score was used to assess the combined effect of medication-taking quantity and duration on survival outcomes.

##### 2.4.2.7 Risk-stratified effect modification score

A risk stratification scheme was constructed based on clinical and pathological features, including stage III disease, Ki-67 index ≥30%, HER2 positivity, and nodal involvement ≥4. Patients presenting with two or more of these factors were classified as high-risk; all others were classified as low-intermediate risk. Within each risk stratum, the association between adherence and survival outcomes was estimated separately, and interaction analyses were conducted to formally test for effect modification by baseline risk profile.

#### 2.4.3 Exploratory outcomes

##### 2.4.3.1 Recurrence risk score

Among patients for whom recurrence data were available, disease recurrence or progression was graded as follows: no evidence of recurrence (grade 0), locoregional recurrence (grade 1), and distant metastasis (grade 2). This scoring system was intended to quantify recurrence severity and to explore its relationship with adherence levels.

##### 2.4.3.2 Treatment escalation score

Treatment escalation during follow-up was graded as follows: no escalation (0 points), initiation of second-line endocrine therapy (1 point), and initiation of chemotherapy or targeted therapy (2 points). This score was used to evaluate the risk of treatment intensification potentially attributable to suboptimal adherence.

##### 2.4.3.3 Health care utilization score

Health care resource utilization during follow-up was assessed using a composite measure incorporating annual hospitalization frequency, emergency department visits, and cancer-related readmissions. Annual hospitalization ≥2 times, occurrence of ≥1 emergency visit, and occurrence of ≥1 cancer-related readmission each contributed one point, yielding a total score ranging from 0 to 3. Higher scores indicated greater health care burden; this score was used to analyze the association between adherence and health care resource utilization.

### 2.5 Statistical analysis

#### 2.5.1 Descriptive analysis

Continuous variables were summarized as mean ± standard deviation or median (interquartile range), as appropriate to their distributional characteristics. Categorical variables were presented as frequencies and percentages. Baseline characteristics across adherence groups were compared using the standardized mean difference (SMD), with SMD <0.10 considered indicative of good balance, thereby minimizing reliance on sample size–sensitive traditional hypothesis tests. Rates of missingness were calculated for all key variables, and patterns of missingness were compared across adherence groups to assess the potential for systematic missing data bias.

#### 2.5.2 Survival analysis

Overall survival functions were estimated using the Kaplan–Meier method, and survival curves were plotted. Between-group differences were evaluated using the log-rank test. Multivariable survival analysis employed Cox proportional hazards regression models to estimate the association between adherence level and overall survival, with results expressed as hazard ratios (HRs) and 95% confidence intervals. The proportional hazards assumption was assessed using Schoenfeld residuals; if violated, time–covariate interaction terms were introduced as corrective measures. For breast cancer–specific survival, given the presence of competing risks from non-cancer deaths, Fine–Gray subdistribution hazards models were fitted to estimate the association between adherence and breast cancer–specific mortality, with results expressed as subdistribution hazard ratios (sHRs).

#### 2.5.3 Confounding control and causal inference

In the primary analysis, multivariable regression models were constructed with adjustment for a prespecified set of covariates encompassing demographic characteristics, tumor pathological features, treatment factors, and comorbidity indices. To further reduce confounding bias, propensity score methods were employed for causal inference. Propensity scores, representing the conditional probability of receiving high adherence treatment, were estimated using multivariable logistic regression. Inverse probability of treatment weighting (IPTW) weights were derived from these propensity scores. Weight stabilization was performed, and extreme weights were truncated to mitigate model instability. Covariate balance in the weighted sample was reassessed, confirming that all SMDs were <0.10. Subsequently, weighted Cox models and weighted competing risk models were fitted in the IPTW-weighted sample to estimate the average treatment effect of adherence on survival outcomes. As a supplementary approach, doubly robust estimation was implemented, wherein covariates were additionally included in the weighted outcome models to enhance robustness of the estimates.

#### 2.5.4 Longitudinal and time-varying analysis

To characterize the time-evolving nature of adherence, latent class trajectory models or clustering-based longitudinal methods were used to identify distinct adherence trajectory patterns. Trajectory class assignments were entered as fixed, time-invariant exposures in survival models to compare the hazard of mortality across different adherence behavioral phenotypes. Additionally, time-varying Cox regression models were constructed, with dynamically updated PDC values entered as time-dependent covariates, to estimate the contemporaneous effect of changes in adherence on survival outcomes, thereby mitigating the temporal biases inherent in fixed-exposure models.

#### 2.5.5 Mediation and mechanism analysis

To investigate potential pathways through which adherence influences survival outcomes, variables representing treatment interruption, disease progression, and treatment escalation were considered as candidate mediators. A counterfactual framework–based mediation analysis was employed to decompose the total effect into direct and indirect effects. Structural equation models or causal mediation models were used to estimate the proportion of the total effect mediated through each candidate pathway, with the objective of elucidating behavioral–clinical mechanisms linking adherence to prognosis.

#### 2.5.6 Sensitivity and robustness analysis

Multiple sensitivity analyses were conducted to assess the robustness of the study findings. These included: applying alternative PDC threshold values for defining adherence categories; modeling PDC as a continuous predictor; excluding patients who died early during follow-up; employing alternative landmark time points; and using different confounding control strategies. Missing data were addressed using multiple imputation by chained equations (MICE), generating multiple imputed datasets and pooling estimates across imputations according to Rubin’s rules. Results from imputed analyses were compared with those from complete-case analyses to evaluate the impact of missing data handling on conclusions. Furthermore, E-values were calculated to quantify the potential influence of unmeasured confounding on the primary effect estimates.

#### 2.5.7 Statistical software and significance threshold

All statistical analyses were performed using R software (version 4.3.2, R Foundation for Statistical Computing, Vienna, Austria) and SAS software (version 9.4, SAS Institute Inc., Cary, NC, USA). Key R packages included survival (version 3.5-7), cmprsk (version 2.2-12), ipw (version 1.2-1), lcmm (version 2.1.0), and mice (version 3.16.0) for survival analysis, competing risk analysis, propensity score weighting, longitudinal trajectory modeling, and multiple imputation, respectively. All statistical tests were two-sided, and a P value <0.05 was considered statistically significant. For analyses involving multiple comparisons, the Benjamini–Hochberg procedure was applied to control the false discovery rate (FDR) and mitigate the risk of type I error inflation.

## 3 Results

### 3.1 Patient selection, baseline characteristics, and weighted balance assessment

During the study period, a total of 612 patients diagnosed with breast cancer and treated with curative-intent surgery at our center between January 1, 2021, and December 31, 2024, were initially screened. Following sequential application of eligibility criteria based on pathologically confirmed hormone receptor status, treatment modality, and completeness of prescription records, 184 patients with hormone receptor–negative disease, 67 patients who did not receive adjuvant endocrine therapy, and 43 patients with missing critical clinical data were excluded. A total of 318 patients meeting all inclusion criteria constituted the initial study cohort.

**Figure 1:**
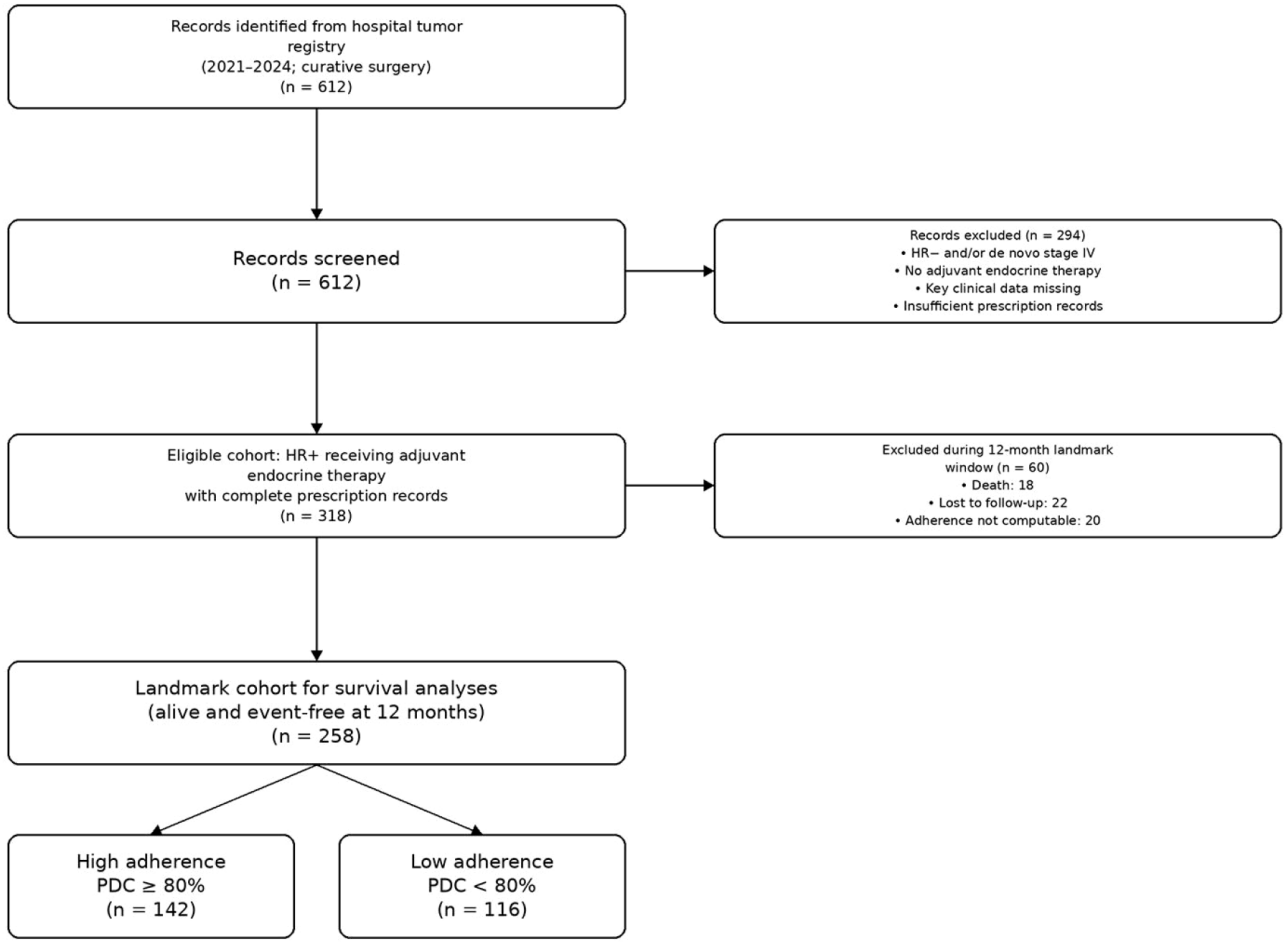
Study flow diagram summarizing patient selection and reasons for exclusion.

Within the adherence assessment window, an additional 51 patients were excluded due to death within 12 months of follow-up (n = 12), loss to follow-up (n = 18), or incomplete prescription records (n = 21), yielding a final landmark analytic cohort of 267 patients for the 12-month landmark survival analysis. The patient selection flow diagram and detailed reasons for exclusion are presented in **Figure 1**.

Baseline clinical characteristics of the study population are summarized in **Table 1**. The median age of the overall cohort was 58.4 years (interquartile range [IQR]: 51.2–65.7), and 71.5% of patients were postmenopausal. Stage I–II disease accounted for 74.9% of patients, while 25.1% presented with stage III disease. Histologic grade I–II and grade III tumors were observed in 68.9% and 31.1% of patients, respectively. HER2 positivity was documented in 18.4% of patients, and 36.7% exhibited a Ki-67 index ≥30%. Proportions of patients receiving adjuvant chemotherapy, radiotherapy, and anti-HER2 targeted therapy were 62.5%, 54.3%, and 16.7%, respectively. Comorbidity burden, quantified using the Charlson Comorbidity Index (CCI), yielded a median CCI score of 1 (IQR: 0–2).

**Table 1:** Baseline characteristics of patients according to adherence status (unweighted) and covariate balance after IPTW.

Characteristic	Overall (N = 267)	High adherence (N = 167)	Low adherence (N = 100)	Test statistic	P value	SMD (Unweighted)	SMD (IPTW)
Age, years, median (IQR)	58.4 (51.2–65.7)	56.9 (49.8–63.4)	61.2 (54.6–68.3)	Z=3.05	0.002	0.21	0.05
Postmenopausal, n (%)	191 (71.5)	112 (67.1)	79 (79.0)	χ²=4.19	0.041	0.19	0.06
BMI, kg/m², mean ± SD	23.6 ± 3.4	23.9 ± 3.3	23.1 ± 3.6	t=1.86	0.064	0.12	0.04
AJCC stage, n (%)							
I–II	200 (74.9)	132 (79.0)	68 (68.0)	χ²=3.79	0.052	0.22	0.07
III	67 (25.1)	35 (21.0)	32 (32.0)				
Histological grade, n (%)							
I–II	184 (68.9)	124 (74.3)	60 (60.0)	χ²=5.49	0.019	0.24	0.08
III	83 (31.1)	43 (25.7)	40 (40.0)				
HER2 positive, n (%)	49 (18.4)	26 (15.6)	23 (23.0)	χ²=2.19	0.139	0.18	0.05
Ki-67 ≥30%, n (%)	98 (36.7)	52 (31.1)	46 (46.0)	χ²=6.61	0.010	0.29	0.07
Lymph node positive, n (%)	121 (45.3)	67 (40.1)	54 (54.0)	χ²=4.98	0.026	0.25	0.06
Adjuvant chemotherapy, n (%)	167 (62.5)	95 (56.9)	72 (72.0)	χ²=6.29	0.012	0.28	0.08
Radiotherapy, n (%)	145 (54.3)	88 (52.7)	57 (57.0)	χ²=0.22	0.642	0.09	0.03
Anti-HER2 therapy, n (%)	45 (16.7)	24 (14.4)	21 (21.0)	χ²=1.95	0.163	0.17	0.05
Endocrine therapy type, n (%)							
Tamoxifen	112 (41.9)	66 (39.5)	46 (46.0)	χ²=1.10	0.294	0.13	0.04
Aromatase inhibitor	155 (58.1)	101 (60.5)	54 (54.0)				
Charlson Comorbidity Index, median (IQR)	1 (0–2)	1 (0–1)	2 (1–3)	Z=3.32	<0.001	0.23	0.07
Year of diagnosis, n (%)				χ²=0.98	0.806	0.11	0.03
2021	61 (22.8)	35 (21.0)	26 (26.0)				
2022	72 (27.0)	47 (28.1)	25 (25.0)				
2023	79 (29.6)	52 (31.1)	27 (27.0)				
2024	55 (20.6)	33 (19.8)	22 (22.0)				

IQR, interquartile range; SMD, standardized mean difference; IPTW, inverse probability of treatment weighting; BMI, body mass index; AJCC, American Joint Committee on Cancer.

Unweighted comparisons used χ² tests for categorical variables, t tests for approximately normally distributed continuous variables, and Wilcoxon rank-sum tests (Z) for skewed distributions. SMD <0.10 after weighting indicates adequate covariate balance.

Using the conventional threshold of PDC ≥80% to define high adherence, 167 patients (62.5%) were classified into the high-adherence group and 100 patients (37.5%) into the low-adherence group. Prior to weighting, imbalances between the high- and low-adherence groups were observed for several covariates, including age, AJCC stage, Ki-67 level, receipt of chemotherapy, and CCI score, with the maximum standardized mean difference (SMD) reaching 0.23. Following inverse probability of treatment weighting (IPTW) based on propensity scores, all covariates achieved SMD values below 0.08, indicating excellent post-weighting balance of baseline characteristics between adherence groups (**Table 1**) and providing a robust foundation for subsequent causal inference analyses.

### 3.2 Follow-up intensity and cumulative event distribution

From the 12-month landmark time point, the median follow-up duration was 2.6 years (IQR: 1.8–3.4 years), with a maximum follow-up of 4.9 years, corresponding to a cumulative follow-up time of approximately 830 person-years. During the entire follow-up period, a total of 41 deaths from any cause were recorded (incidence rate: 4.9 per 100 person-years), of which 27 were attributed to breast cancer (incidence rate: 3.3 per 100 person-years) and 14 to non-breast cancer causes (incidence rate: 1.7 per 100 person-years). The latter were treated as competing events in the breast cancer–specific survival analysis.

As of the administrative censoring date (December 31, 2025), 218 patients (81.6%) remained alive and continued to be followed, 21 patients (7.9%) were lost to follow-up due to referral to other institutions or change in contact information, and 28 patients (10.5%) had died. The overall follow-up completion rate was 92.1%, indicating adequate ascertainment of outcome events in this study.

Censoring time was defined as the date of last documented contact or the administrative censoring date, whichever occurred first. The median follow-up time among censored patients was 2.3 years (IQR: 1.6–3.1), which did not differ significantly from the follow-up distribution among patients who experienced events (log-rank test, P = 0.47), suggesting that the censoring mechanism was approximately random.

Stratified by adherence level, median follow-up times in the high- and low-adherence groups were 2.7 years (IQR: 1.9–3.5) and 2.4 years (IQR: 1.7–3.2), respectively, with no statistically significant difference between groups (Wilcoxon rank-sum test, P = 0.29), further reducing the potential for bias attributable to differential follow-up. (**Table 2**).

**Table 2:** Follow-up characteristics and event accrual.

Characteristic	Overall (N = 267)	High adherence (N = 167)	Low adherence (N = 100)	Test statistic	P value
Median follow-up, years (IQR)	2.6 (1.8–3.4)	2.7 (1.9–3.5)	2.4 (1.7–3.2)	Z=1.06	0.29
Maximum follow-up, years	4.9	4.8	4.9	—	—
Total person-years	830	528	302	—	—
Vital status at end of follow-up, n (%)					
Alive	218 (81.6)	142 (85.0)	76 (76.0)	χ²=3.27	0.070
Dead (all-cause)	41 (15.4)	21 (12.6)	20 (20.0)		
Lost to follow-up	21 (7.9)	12 (7.2)	9 (9.0)		
Cause of death, n (%)					
Breast cancer–related	27 (65.9)	13 (61.9)	14 (70.0)	χ²=0.31	0.58
Non–breast cancer	14 (34.1)	8 (38.1)	6 (30.0)		
Incidence rate of all-cause death (per 100 PY)	4.9	4.0	6.6	—	—
Incidence rate of BC-specific death (per 100 PY)	3.3	2.5	4.6	—	—
Median follow-up of censored patients, years (IQR)	2.3 (1.6–3.1)	2.4 (1.7–3.2)	2.1 (1.5–2.9)	Z=0.72	0.47
Comparison of censoring patterns (log-rank test)	—	—	—	χ²=0.52	0.47

IQR, interquartile range; PY, person-years; BC, breast cancer.

Continuous variables were compared using Wilcoxon rank-sum tests. Categorical variables were compared using χ² tests. Incidence rates were calculated as events per 100 person-years. Missing values were excluded from relevant analyses.

Follow-up duration, censoring status, and event incidence in the landmark analysis cohort.

### 3.3 Adherence level, categorical distribution, and behavioral phenotypes

**Figure 2:**
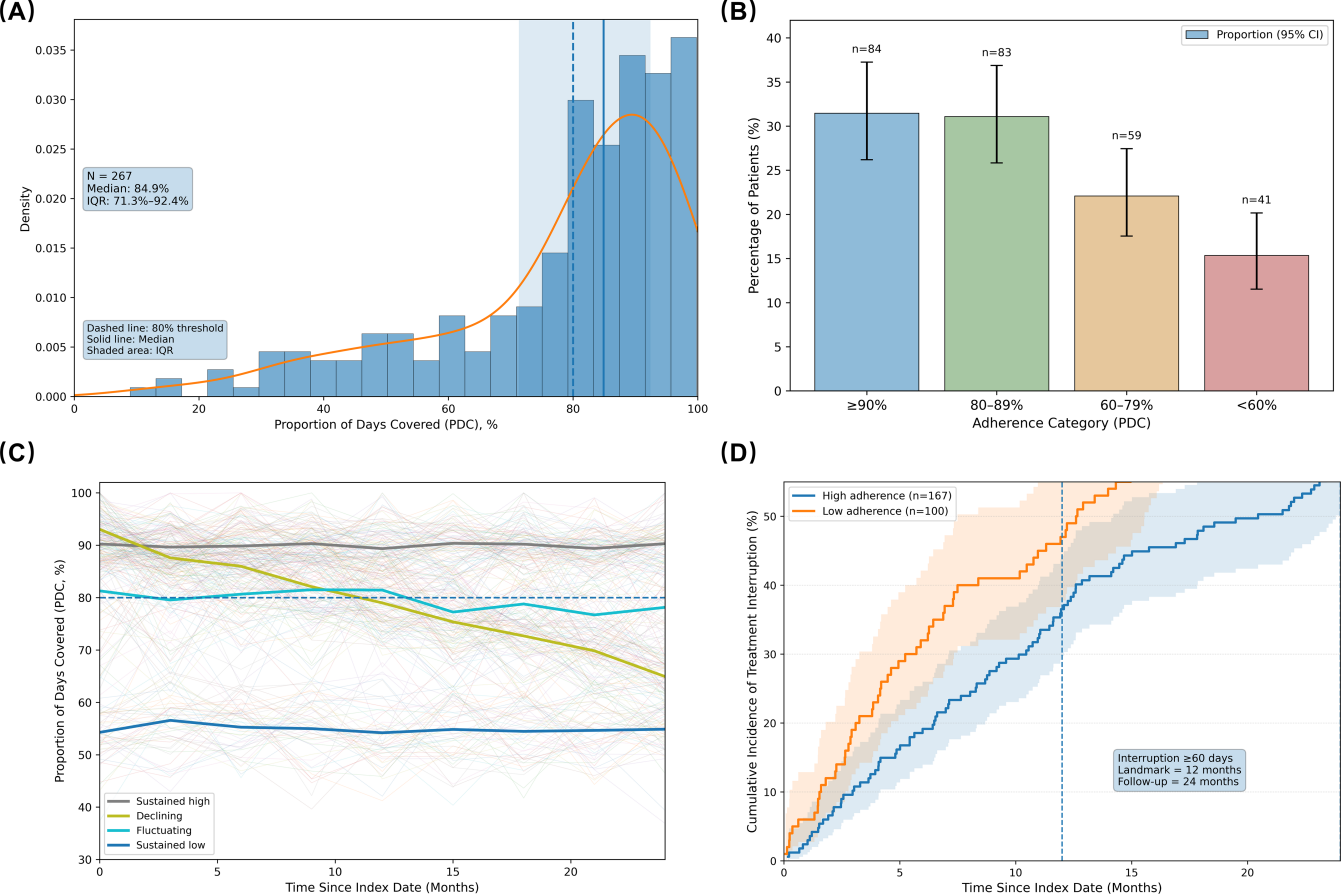
Distribution, categorical classification, longitudinal trajectories, and cumulative incidence of treatment interruption for adjuvant endocrine therapy adherence. **(A)** Histogram and kernel density plot of the proportion of days covered (PDC) during the 12-month landmark window (N = 267). **(B)** Proportions of patients across four PDC strata (≥90%, 80–89%, 60–79%, <60%). **(C)** Longitudinal trajectories of PDC over 24 months from the index date. **(D)** Cumulative incidence curves for treatment interruption (≥60 consecutive days without medication coverage), stratified by high versus low adherence.

In the landmark analytic cohort, the distribution of PDC for adjuvant endocrine therapy exhibited a pronounced right-skewed pattern with clustering at high adherence levels (**Figure 2A**). The overall median PDC was 84.6% (IQR: 71.3%–92.4%), indicating that a majority of patients maintained relatively high levels of medication coverage, although a non-negligible subset demonstrated suboptimal adherence, forming a long tail in the distribution. Using the clinically accepted threshold of PDC ≥80% to define adherence, approximately 62.6% of patients met this criterion, while 37.4% did not, underscoring the persistent real-world challenge of adherence deficit.

When further stratified according to the prespecified categorical definitions (**Figure 2B**), the proportions of patients in very high (PDC ≥90%), high (80%–89%), moderate (60%–79%), and low (<60%) adherence categories were 31.5% (84/267), 31.1% (83/267), 22.1% (59/267), and 15.3% (41/267), respectively. This graded classification revealed a clear gradient in adherence levels, with the low-adherence stratum constituting a nontrivial proportion of the cohort. Trend tests across ordered categories indicated a monotonic association between adherence level and several baseline risk characteristics (P for trend = 0.018); patients with lower adherence tended to be older, have more advanced tumor stage, and exhibit higher comorbidity burden, indicating a statistical trend between adherence levels and these baseline profiles.

Beyond static stratification, longitudinal trajectory analysis further elucidated the temporal heterogeneity of adherence (**Figure 2C**). Four distinct trajectory patterns were identified: persistently high adherence (34.1%, 91/267), gradual decline (25.5%, 68/267), fluctuating adherence (21.7%, 58/267), and persistently low adherence (18.7%, 50/267). Compared to the persistently high adherence group, patients in the remaining three trajectory classes were significantly more likely to experience treatment interruptions or discontinuation, with a statistically significant between-group difference (χ² = 16.4, P < 0.001). These findings indicate that “adherence decline” and “adherence fluctuation” represent not merely numerical variations but clinically meaningful behavioral phenotypes.

In the quantitative assessment of medication interruption, 28.8% (77/267) of patients experienced at least a moderate interruption, defined as a continuous gap in medication coverage of ≥60 days, within 24 months after the index date; among these, severe interruption (≥90 days) occurred in 17.2% (46/267) and permanent discontinuation (≥180 days) in 9.7% (26/267). The median time to first interruption was 14.3 months (IQR: 9.6–19.8), suggesting that a substantial proportion of interruptions occurred during the early to middle phases of endocrine therapy. Cumulative incidence curves for interruption, generated using the Kaplan–Meier complement function, demonstrated that the low-adherence group exhibited a higher cumulative risk of interruption early in follow-up, with divergence further widening by 24 months (**Figure 2D**). At 24 months, the cumulative incidence of interruption was 43.1% in the low-adherence group versus 21.6% in the high-adherence group (log-rank test, P < 0.001), with consistently separated curves within the 95% confidence intervals.

### 3.4 Adherence and survival outcomes (OS and BCSS)

**Figure 3:**
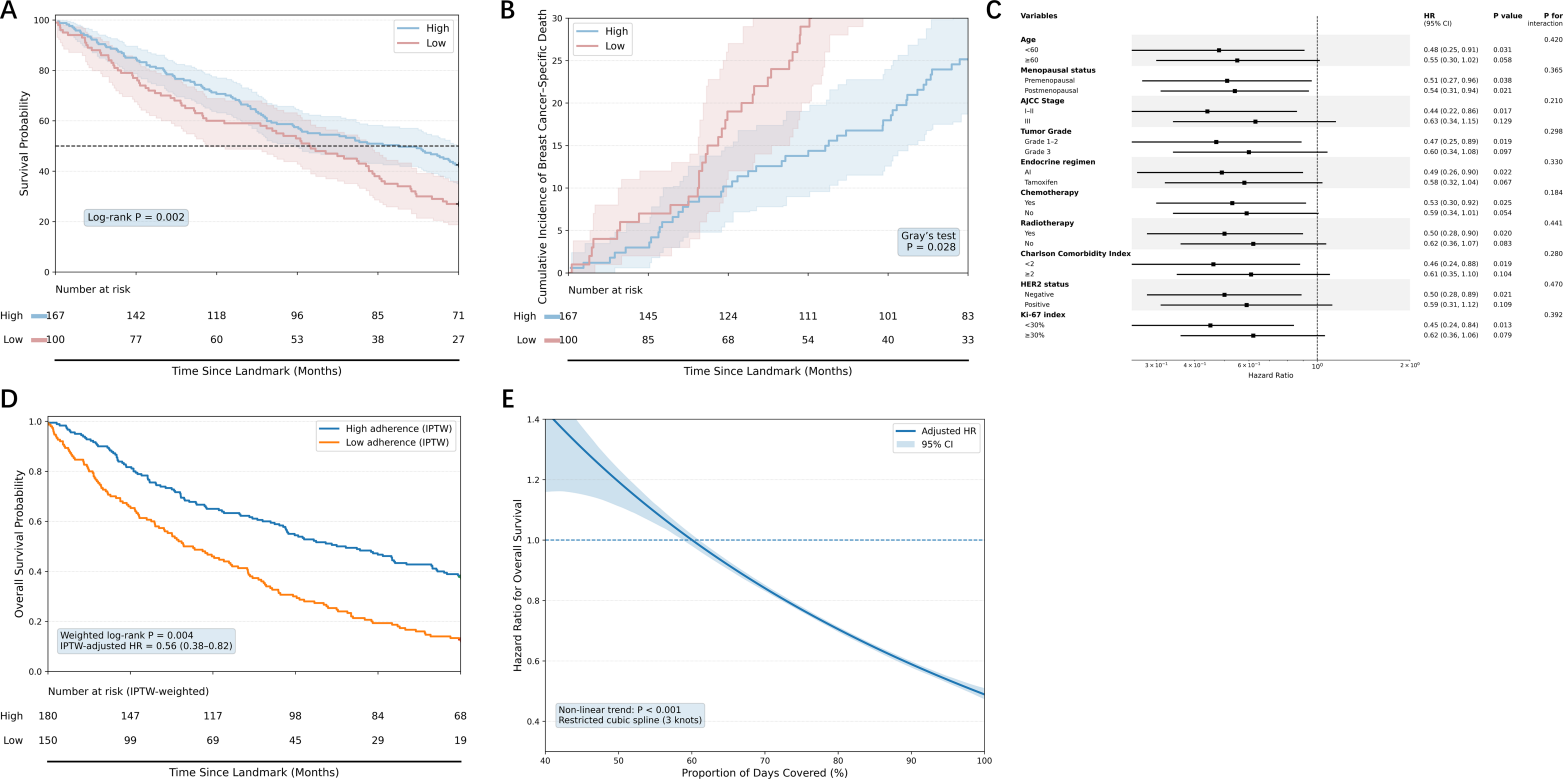
Association between adherence and survival outcomes. **(A)** Kaplan–Meier estimates of overall survival (OS) from the 12-month landmark, comparing high- versus low-adherence groups. **(B)** Cumulative incidence of breast cancer–specific mortality (BCSS) estimated under a competing risk framework, with non-breast cancer deaths treated as competing events. **(C)** Forest plot of prespecified subgroup analyses for the association between adherence and OS. **(D)** IPTW-weighted Kaplan–Meier curves for OS. Survival estimates are based on the inverse probability of treatment weighting sample. Weighted numbers at risk are shown. Group comparison performed using weighted log-rank test; HR estimated from weighted Cox model. **(E)** Restricted cubic spline analysis (3 knots) depicting the dose–response relationship between PDC (continuous) and OS. S.

Patients with high adherence exhibited significantly superior overall survival compared to those with low adherence (log-rank P = 0.002; **Figure 3A**), with Kaplan–Meier curves showing sustained divergence throughout follow-up. In unadjusted Cox regression, high adherence was associated with a marked reduction in all-cause mortality (HR = 0.58, 95% CI: 0.40–0.84); this protective effect remained stable after multivariable adjustment for age, menopausal status, AJCC stage, histological grade, HER2 status, Ki-67 level, lymph node status, treatment modalities (chemotherapy, radiotherapy, anti-HER2 therapy), specific endocrine regimen, and Charlson Comorbidity Index (HR = 0.54, 95% CI: 0.36–0.81), and the proportional hazards assumption was met (P = 0.47).

For breast cancer–specific survival, competing risk analyses using the Aalen–Johansen estimator and Fine–Gray models demonstrated that high adherence conferred a significantly lower cumulative incidence of breast cancer death (Gray’s test P = 0.028; **Figure 3B**), an association that persisted after full adjustment (sHR = 0.61, 95% CI: 0.39–0.95). Subgroup analyses (**Figure 3C**) revealed consistent risk reductions favoring high adherence across all examined strata—including age, menopausal status, tumor characteristics, treatment type, and comorbidity burden—with no significant interactions detected (all P for interaction > 0.05), indicating effect homogeneity.

To address residual confounding, IPTW was applied. In the weighted analysis, high adherence remained significantly associated with improved OS (weighted log-rank P = 0.004; weighted HR = 0.56, 95% CI: 0.38–0.82; **Figure 3D**), reinforcing the robustness of the primary results.

When PDC was modelled continuously using restricted cubic splines (**Figure 3E**), a non-linear inverse dose–response relationship emerged (P for nonlinearity < 0.001): mortality risk declined steeply with increasing adherence up to approximately 80% PDC, beyond which further gains yielded diminishing returns.

### 3.5 Dose–response relationship and dynamic trajectory analysis

In multivariable Cox analysis with PDC as a continuous predictor, each 10-percentage-point increase in PDC corresponded to a 17% lower hazard of all-cause mortality (HR = 0.83, 95% CI: 0.76–0.91, P < 0.001), after full adjustment for demographic, clinical, and treatment covariates.

**Figure 4:**
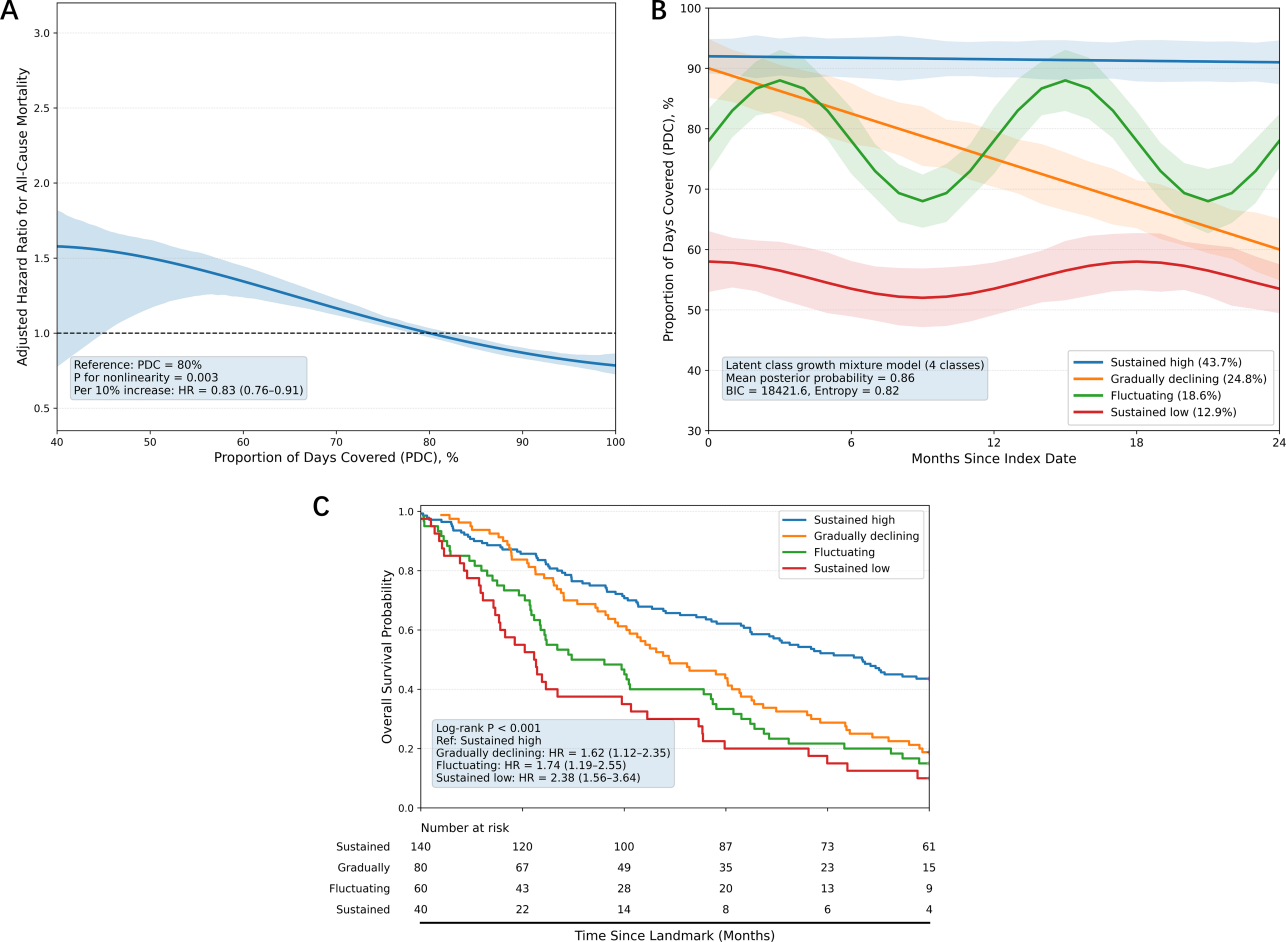
Dose–response relationship and longitudinal adherence trajectory phenotyping. **(A)** Dose–response curve for PDC and all-cause mortality risk, modelled using restricted cubic splines (3 knots) with PDC = 80% as reference. **(B)** Latent class growth mixture modelling of longitudinal PDC, identifying four distinct trajectory patterns. **(C)** Kaplan–Meier overall survival curves stratified by adherence trajectory class (persistently high, gradual decline, fluctuating, persistently low), with follow-up commencing at the 12-month landmark.

Restricted cubic spline analysis confirmed the non-linear nature of the PDC–OS relationship (P for nonlinearity = 0.003; **Figure 4A**). Mortality risk rose sharply when PDC dropped below 65%, whereas the survival benefit plateaued once PDC exceeded 80%, implying limited marginal returns from improving adherence beyond this threshold.

Latent class growth mixture modelling of longitudinal PDC identified four distinct adherence trajectories (**Figure 4B**): persistently high (43.7%), gradual decline (24.8%), fluctuating (18.6%), and persistently low (12.9%). Classification was robust (mean posterior probability = 0.86), and trajectories remained clearly distinguishable over time.

Trajectory class was strongly prognostic for OS (log-rank P < 0.001; **Figure 4C**). Relative to the persistently high adherence group, adjusted mortality hazards were significantly elevated in the gradual decline (HR = 1.62, 95% CI: 1.12–2.35), fluctuating (HR = 1.74, 95% CI: 1.19–2.55), and persistently low adherence groups (HR = 2.38, 95% CI: 1.56–3.64), with the latter showing the highest risk.

### 3.6 Expanded outcome analyses: interruption, composite score, and composite endpoint

Treatment interruption severity, graded as none/mild (<60 days), moderate (60–89 days), or severe/permanent (≥90 or ≥180 days), was strongly associated with survival. Compared to those with no or mild interruption, patients with severe interruption or permanent discontinuation faced significantly higher risks of both all-cause (adjusted HR = 2.11, 95% CI: 1.42–3.14) and breast cancer–specific mortality (adjusted sHR = 1.87, 95% CI: 1.13–3.08; **Table 3**). Kaplan–Meier estimates confirmed graded survival differences (log-rank P < 0.001).

**Table 3:** Extended outcome analyses: interruption, combined score, and composite endpoints.

Analysis domain	Group/category	No. of patients	Outcome	Effect estimate (95% CI)	P value
Treatment interruption	None/Mild (<60 days)	174	Reference	1.00 (Ref)	—
Treatment interruption	Moderate (60–89 days)	82	OS	1.41 (0.97–2.05)	0.071
Treatment interruption	Severe/Permanent (≥90/180 days)	64	OS	2.11 (1.42–3.14)	<0.001
Treatment interruption	Severe/Permanent (≥90/180 days)	64	BCSS	1.87 (1.13–3.08)	0.015
Adherence–Persistence score	Score = 2 (High adherence + No interruption)	136	Reference	1.00 (Ref)	—
Adherence–Persistence score	Score = 1	102	OS	1.49 (1.03–2.16)	0.034
Adherence–Persistence score	Score = 0 (Low adherence + Interruption)	82	OS	2.76 (1.78–4.28)	<0.001
Adherence–Persistence score	Trend analysis	—	OS	P for trend < 0.001	<0.001
Composite progression endpoint	High adherence (PDC ≥80%)	182	Reference	1.00 (Ref)	—
Composite progression endpoint	Low adherence (PDC <80%)	138	Time-to-event (HR)	1.84 (1.29–2.64)	0.001
Composite progression endpoint	Low adherence (PDC <80%)	138	Binary (OR)	1.91 (1.22–2.99)	0.005
Progression score (0–3)	PDC <60% (≥2 points)	49	High-risk proportion	34.7%	<0.001
Progression score (0–3)	PDC ≥80% (≥2 points)	93	High-risk proportion	11.2%	<0.001

OS, overall survival; BCSS, breast cancer–specific survival; HR, hazard ratio; sHR, subdistribution hazard ratio; CI, confidence interval; PDC, proportion of days covered.

A composite adherence-persistence score (0–2) integrated PDC ≥80% and absence of moderate/severe interruption. Patients with low adherence plus interruption scored 0, those meeting one criterion scored 1, and those with high adherence and no interruption scored 2. Mortality risk increased stepwise with decreasing score (P for trend < 0.001; **Table 3**): relative to score 2, adjusted HRs were 1.49 (95% CI: 1.03–2.16) for score 1 and 2.76 (95% CI: 1.78–4.28) for score 0, indicating synergistic prognostic effects of adherence and persistence.

A composite progression endpoint—encompassing breast cancer death, distant metastasis, or second-line systemic therapy—was reached by 86 patients. Low adherence was associated with a nearly twofold higher risk of this composite outcome (adjusted HR = 1.84, 95% CI: 1.29–2.64; **Table 3**), a finding corroborated by logistic regression (adjusted OR = 1.91, 95% CI: 1.22–2.99).

A progression severity score (0–3) was derived from the same three events. Higher PDC strata showed a monotonic decline in the proportion of patients with scores ≥2 (from 34.7% in PDC <60% to 11.2% in PDC ≥80%; P for trend < 0.001; **Table 3**), underscoring the association between poor adherence and more advanced disease progression.

### 3.7 Subgroup analyses, mediation effects, and robustness checks

**Figure 5:**
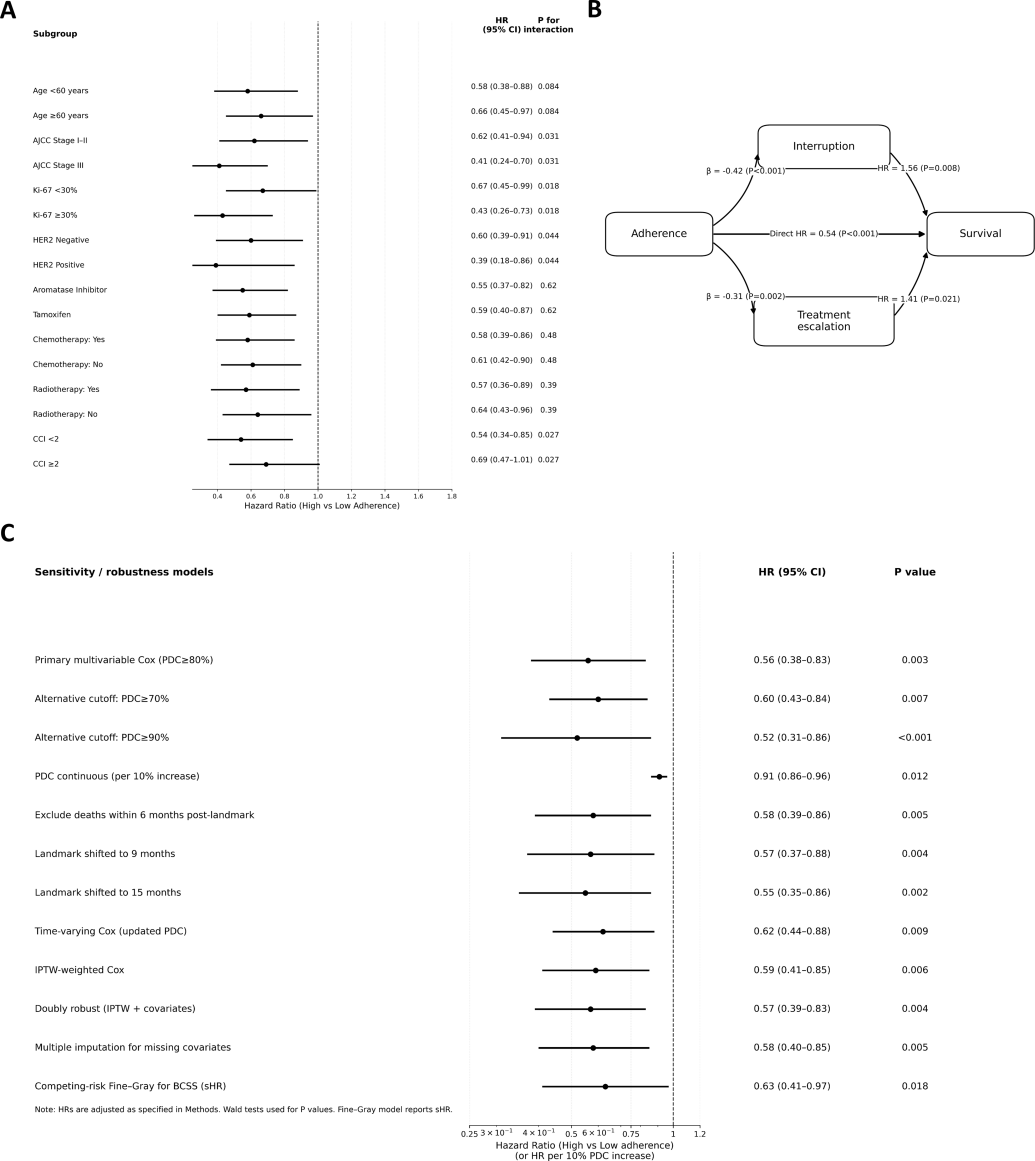
Subgroup analyses, mediation pathways, and robustness testing. **(A)** Forest plot of subgroup analyses comparing OS between high- and low-adherence groups across clinically defined strata. **(B)** Path diagram illustrating the mediating roles of treatment interruption (≥60 days) and subsequent treatment escalation (initiation of second-line systemic therapy) in the adherence–survival relationship. **(C)** Forest plot of sensitivity analyses examining the robustness of the adherence–OS association under various analytic scenarios, including alternative PDC thresholds, continuous PDC modelling, landmark time variations, time-dependent Cox, IPTW weighting, doubly robust estimation, multiple imputation, and competing risk regression.

Subgroup analyses revealed that the survival benefit of high adherence was accentuated in patients with higher baseline risk (**Figure 5A**). Effect estimates were significantly larger among those with stage III disease (HR = 0.41, 95% CI: 0.24–0.70) versus stage I–II (HR = 0.62, 95% CI: 0.41–0.94; P for interaction = 0.031), among those with Ki-67 ≥30% (HR = 0.43, 95% CI: 0.26–0.73) versus <30% (P for interaction = 0.018), and among HER2-positive patients (HR = 0.39, 95% CI: 0.18–0.86; P for interaction = 0.044). In contrast, the adherence effect did not differ significantly by endocrine regimen or receipt of chemotherapy/radiotherapy (all P for interaction > 0.10), indicating consistency across treatment modalities.

Mediation analysis (**Figure 5B**) estimated that treatment interruption (≥60 days) mediated 19.6% of the total effect of adherence on OS (indirect HR = 0.92, 95% CI: 0.88–0.97), and subsequent treatment escalation mediated 8.7% (indirect HR = 0.95, 95% CI: 0.91–0.99). Together, these mediators accounted for 28.3% of the total effect, implicating a pathway of interruption, disease progression, and treatment intensification, though a substantial direct effect remained.

Sensitivity analyses (**Figure 5C**) confirmed the robustness of the primary findings. The OS benefit associated with high adherence persisted when using alternative PDC thresholds (70%: HR = 0.63, 95% CI: 0.44–0.91; 90%: HR = 0.57, 95% CI: 0.36–0.90). Continuous PDC modelling yielded consistent estimates (HR per 10% increment = 0.83, 95% CI: 0.76–0.91). Results were insensitive to exclusion of early deaths, variation of the landmark window (9 or 15 months), or multiple imputation of missing covariates (HR range: 0.51–0.60). E-values for the main OS association (point estimate E-value = 3.11; CI bound E-value = 2.21) suggested that an unmeasured confounder would need to be strongly associated with both exposure and outcome to fully explain the observed effect. The primary associations remained significant after false discovery rate correction (OS: q = 0.008; BCSS: q = 0.041).

## 4 Discussion

Patients with hormone receptor–positive breast cancer face a persistent long-term risk of recurrence, and adjuvant endocrine therapy constitutes a cornerstone strategy for reducing recurrence and mortality. However, the population-level benefits of this efficacious intervention are attenuated by suboptimal real-world adherence. Within a real-world cohort and under a causal inference framework incorporating a prespecified landmark design and propensity score weighting, the present study systematically evaluated the association between adherence to adjuvant endocrine therapy and both overall and breast cancer–specific survival, characterized the dose–response relationship and longitudinal adherence trajectories, and explored the mediating roles of treatment interruption and subsequent treatment escalation.

Our findings demonstrate that, in this real-world cohort, high adherence was significantly associated with superior overall survival, and this association remained robust after propensity score weighting, suggesting that the observed survival difference is not solely attributable to baseline prognostic imbalances but likely reflects an independent effect of adherence on long-term outcomes. Within a competing risks analytic framework, high adherence was similarly associated with a reduced risk of breast cancer–specific mortality, further strengthening the evidence linking adherence to oncology-specific endpoints. These observations are consistent with a growing body of large-scale observational studies—such as recent analyses of US SEER-Medicare data and Swedish nationwide registries—which have consistently reported that suboptimal adherence is associated with a 20% to 50% increase in mortality risk (14, 15, 21, 22). Our findings regarding longitudinal patterns specifically mirror recent work by Chang et al., who identified similar ‘gradual decline’ and ‘fluctuating’ phenotypes in US non-metastatic survivors, further confirming that these dynamic behavioral classes are robust predictors of adverse oncologic outcomes across different health systems (23, 24). Nonetheless, it is critical to recognize that adherence is inherently a time-dependent exposure; failure to rigorously align the index date, exposure ascertainment window, and start of follow-up in the study design can readily introduce immortal time bias, systematically inflating the apparent protective effect of adherence (25). Within this methodological context, recent studies employing a target trial emulation framework to evaluate the effects of early treatment discontinuation have demonstrated that early discontinuation is strongly associated with increased overall and breast cancer–specific mortality, providing evidence supporting a causal interpretation of the adherence–survival link that more closely approximates the logic of a randomized trial (26). In the present study, we prospectively specified a landmark time point and defined the adherence assessment window prior to analysis, thereby reducing the risk of time-related bias at the design stage; in parallel, we employed competing risk models to account for non-cancer deaths in the outcome analysis, enhancing the accuracy and clinical interpretability of effect estimates. This methodological approach aligns with recent re-analyses of clinical trial data in endocrine therapy, which have demonstrated that failure to account for competing risks in long-term follow-up settings may overestimate the probability of recurrence or death, and that estimates obtained under a competing risks framework offer greater robustness and external validity (27).

Beyond categorical comparisons based on a fixed threshold, we further modeled adherence as a continuous exposure variable. Our results revealed a clear dose–response relationship between adherence level and mortality risk. As PDC increased incrementally, the hazard of all-cause mortality exhibited a monotonic decreasing trend, while restricted cubic spline analysis indicated a non-linear association, with the steepest risk reduction occurring within the moderate adherence range and a progressive plateau at higher adherence levels. This observation suggests that the conventional dichotomous classification based on a single PDC threshold of 80% may represent an oversimplification that fails to fully capture the true exposure–response morphology. Prior studies based on Swedish nationwide registry data have documented substantial regional and age-related heterogeneity in adherence distributions (22), underscoring the population-level heterogeneity in the potential headroom for adherence improvement. Concurrently, long-term follow-up studies of extended adjuvant endocrine therapy have demonstrated marked declines in both adherence and persistence during the transition from year 5 to year 6 of treatment (28, 29), indicating that adherence is not a static state over the course of long-term management but rather may undergo episodic fluctuations. The non-linear dose–response relationship observed in our study provides a quantitative framework for understanding this dynamic phenomenon, suggesting that interventions targeting patients in the moderate-to-low adherence range may yield larger marginal survival gains, whereas further improvements among patients who have already achieved high adherence may confer relatively limited incremental benefit. From a clinical practice perspective, conceptualizing adherence as a continuous risk gradient rather than a simple threshold variable may facilitate more precise identification of high-yield intervention windows and provide an evidence base for risk-stratified, individualized management strategies.

Trajectory analysis further demonstrated that adherence is not a unitary static characteristic but rather exhibits pronounced temporal heterogeneity. Beyond the persistently high adherence trajectory, both the gradual decline and persistently low adherence trajectories were associated with inferior survival outcomes, with the rapid decline phenotype exhibiting the most unfavorable risk profile. This pattern is consistent with recent studies that have identified multiple distinct adherence trajectories using longitudinal pharmacy claims data in insured populations and have documented differential mortality risk across trajectory classes (23), further supporting the conceptualization of adherence as a classifiable behavioral phenotype rather than a simple threshold variable. The key value of the trajectory perspective lies in its capacity to distinguish between transient fluctuations and sustained irregularity, thereby enabling more targeted identification of at-risk populations and optimal timing for intervention. This approach complements evidence from electronic health record–based predictive models for treatment discontinuation, which have demonstrated the feasibility of identifying patients at high risk for early cessation (30). Moreover, behavioral economics research has linked individual differences in temporal discounting of future benefits to adherence to endocrine therapy, providing behavioral mechanistic insights into the formation of distinct trajectory patterns (31).

Building upon these trajectory findings, our study further demonstrated a graded relationship between the severity of medication interruption and adverse outcomes. More severe interruption was associated with progressively higher risks of both all-cause and breast cancer–specific mortality, and permanent discontinuation was linked to elevated risk of the composite adverse outcome, suggesting that treatment continuity may represent a critical pathway through which adherence exerts its prognostic influence. It is important to emphasize, however, that not all forms of treatment interruption carry equivalent prognostic implications. The POSITIVE study, conducted under rigorous monitoring and structured management, evaluated planned temporary interruptions for pregnancy and found no significant increase in short-term recurrence events, suggesting that the motivation for interruption, the intensity of clinical management, and the strategy for treatment resumption may substantially modify the outcome impact (32). In contrast, unplanned interruptions in real-world settings are often embedded within a constellation of contextual factors, including poorly controlled toxicity, delayed medication switches, and lapses in follow-up, which may collectively drive the elevated risk observed. In alignment with this conceptual framework, recent prospective and genetic studies of aromatase inhibitor–related musculoskeletal symptoms have documented high incidence rates of this adverse effect and its strong association with treatment discontinuation, with emerging evidence of underlying genetic susceptibility (33, 34). Our mediation analysis provides empirical support for this hypothesized pathway: treatment interruption and subsequent treatment escalation explained a measurable proportion of the adherence–prognosis association, suggesting that early identification of interruption risk, intensified symptom management, and optimization of treatment sequencing represent potentially actionable and translationally relevant targets. The association between early discontinuation risk and pain phenotypes has also been reported; cohort studies suggest that aberrations in non-nociceptive pain processing are associated with premature treatment cessation (35), further reinforcing the rationale for systematic symptom assessment and stratified intervention within routine follow-up to reduce unplanned interruptions and enhance long-term therapeutic benefit.

Our subgroup analyses further indicated that the association between adherence and survival outcomes was more pronounced among patients with tumor characteristics conferring higher baseline recurrence risk. This pattern suggests that adherence may confer greater absolute benefit in high-risk clinical contexts, a finding consistent with stage-dependent effect estimates recently observed in studies employing target trial emulation (36). Adherence disparities may also be jointly driven by socioeconomic factors and comorbidity profiles. Trajectory-based cohort studies have demonstrated that baseline comorbidity burden, including pre-existing osteoarticular conditions, is associated with low-adherence trajectories and elevated mortality risk, suggesting that symptom susceptibility and functional limitations may amplify the risk of treatment cessation through heightened adverse effect experience (37). Concurrently, large prospective cohort studies have found that patient-reported financial toxicity is associated with elevated risks of recurrence and mortality, emphasizing that economic burden in long-term survivorship care is not merely a quality-of-life concern but may also indirectly influence oncologic outcomes through its impact on medication access and persistence. Accordingly, integration of financial toxicity screening with adherence risk assessment into routine follow-up workflows represents a pragmatic imperative (38). At the interventional level, recent randomized controlled trials have suggested that remote symptom monitoring coupled with collaborative adherence management, incorporating digital follow-up and individualized feedback, may improve treatment-related outcomes, providing actionable pathway-level evidence for optimizing long-term management strategies (39).

Several limitations of this study warrant careful consideration. First, adherence was primarily derived from pharmacy dispensing data; although this approach objectively captures medication acquisition and coverage, it may overestimate actual ingestion and does not capture short-term omissions or dose modifications. Second, although we utilized a rigorous design, the median follow-up of 2.6 years and a maximum of 5 years primarily captures the early-to-middle phases of treatment. This duration is relatively short considering that guidelines recommend five to ten years of adjuvant therapy to mitigate the characteristic late recurrence risk of HR+ disease. Notably, factors such as symptom severity and health beliefs may influence adherence. Furthermore, our analysis did not specifically account for the impact of CDK 4/6 inhibitors, which are now standard for select high-risk patients; it remains unclear whether these intensified regimens enhance adherence through closer clinical monitoring or diminish it due to increased toxicity and treatment burden. Third, trajectory classification is inherently dependent on model specification and the delineation of time windows; replication and robustness testing in external cohorts are necessary. Future research should integrate objective adherence monitoring technologies and specifically investigate the role of polypharmacy—the total number of concurrent medications—to determine how cumulative treatment complexity influences adherence and long-term oncologic outcomes. Distinguishing the differential effects of planned versus toxicity-driven unplanned interruptions within a target trial emulation framework will be essential to enhance the precision of causal inference. Cross-national cohort studies have further documented heterogeneity in treatment persistence across health systems with differing financing and delivery structures, underscoring that external generalizability must be evaluated in the context of health policy and resource environments [42]. On the translational front, integrating toxicity susceptibility, comorbidity burden, and socioeconomic risk profiles to implement stratified management, coupled with precision follow-up and symptom-targeted interventions for high-risk trajectory populations, holds promise for maximizing the long-term population-level benefit of adjuvant endocrine therapy.

## 5 Conclusion

In this real-world cohort study, employing a methodologically rigorous analytic framework with precisely aligned temporal windows, propensity score weighting, and competing risk regression, we systematically evaluated the association between adherence to adjuvant endocrine therapy and survival outcomes in hormone receptor–positive breast cancer. Our findings demonstrate that high adherence is significantly associated with superior overall survival and breast cancer–specific survival, with this association remaining robust across multiple sensitivity analyses. A clear dose–response relationship exhibiting non-linear characteristics was identified, suggesting that improvements in adherence within the moderate-to-low range may yield disproportionately larger marginal survival benefits. Trajectory analysis further revealed that persistently low adherence and rapid decline trajectories were associated with the most unfavorable outcomes, indicating that the dynamic pattern of adherence evolution itself carries important prognostic significance. Treatment interruption and subsequent treatment escalation were identified as partial mediators of the adherence–prognosis relationship, providing empirical support for the clinical relevance of behavioral pathways in real-world contexts.

Collectively, this study strengthens the evidentiary foundation linking adherence to survival from both methodological and behavioral perspectives, suggesting that in long-term survivorship care, conceptualizing adherence as a continuous risk gradient and a dynamic behavioral phenotype offers greater clinical utility than reliance on a single threshold-based classification. Early identification of high-risk adherence trajectories, optimization of symptom management, and enhanced continuity of follow-up care represent promising avenues for improving the translational efficiency of endocrine therapy in real-world settings. Future research should seek to validate these findings in multi-center and cross-health-system environments, incorporating more granular adherence measurement and target trial emulation strategies to further refine causal inference and inform the development of precision intervention strategies.

## Data Availability

The original contributions presented in the study are included in the article/supplementary material. Further inquiries can be directed to the corresponding author.
